# Analysis of Myocardial Textures in Relation to Nicotine Abuse Using Radiomics in Cardiac PCCT

**DOI:** 10.3390/tomography12060081

**Published:** 2026-06-01

**Authors:** Felix Waßmer, Rouven Bauer, Stefan O. Schoenberg, Alexander Hertel, Isabelle Ayx

**Affiliations:** Department of Radiology and Nuclear Medicine, University Medical Center Mannheim, Heidelberg University, Theodor-Kutzer-Ufer 1-3, 68167 Mannheim, Germany; rouven.bauer01@stud.uni-heidelberg.de (R.B.); stefan.schoenberg@umm.de (S.O.S.); alexander.hertel@umm.de (A.H.); isabelle.ayx@medma.uni-heidelberg.de (I.A.)

**Keywords:** photon-counting CT, radiomics, myocardial tissue characterization, nicotine status, machine learning

## Abstract

**Background/Objectives**: Photon-counting computed tomography (PCCT) combined with radiomics enables advanced myocardial tissue characterization beyond conventional imaging. This study investigated whether myocardial radiomic features derived from PCCT are associated with nicotine status in patients without coronary artery disease. **Methods**: In this retrospective, single-center study, 104 patients (38 men, 66 women; median age 54 years) without coronary calcification (Agatston score = 0) underwent cardiac PCCT. Myocardial septal thickness was measured at three points during the 65–70% cardiac phase. Myocardial tissue was manually segmented, and 105 radiomic features were extracted. After correlation-based feature reduction, 45 independent features were used for analysis. Patients were categorized based on nicotine status. Machine learning models, including logistic regression, random forest, and gradient boosting, were trained and evaluated using stratified five-fold cross-validation. Model performance was assessed using the area under the receiver operating characteristic curve (ROC-AUC) and additional classification metrics. **Results**: No significant differences in myocardial septal thickness were observed between smokers and non-smokers (*p* > 0.05). However, radiomic features enabled moderate discrimination between smokers and non-smokers. Logistic regression with L2 regularization achieved the best performance (ROC-AUC 0.66, balanced accuracy 0.67), outperforming random forest and gradient boosting models. The most relevant radiomic features primarily comprised higher-order texture and shape-based parameters associated with spatial gray-level heterogeneity and subtle variations in myocardial tissue architecture. **Conclusions**: PCCT-based radiomics may capture subtle myocardial imaging signatures associated with smoking status, even in the absence of structural changes detectable by conventional metrics. These findings highlight the potential of cardiac radiomics as a non-invasive imaging biomarker for early cardiovascular risk assessment and support its integration into advanced cardiac imaging workflows. Future multicenter studies with larger cohorts, external validation, and multimodal correlation are warranted to improve robustness and facilitate clinical translation.

## Simple Summary

Smoking can harm the heart before visible changes appear on standard scans. Using advanced computed tomography with radiomics analysis, this study detected subtle heart muscle differences associated with nicotine status despite normal findings. These results highlight the potential of this approach for early risk detection and future personalized cardiovascular care.

## 1. Introduction

Advances in cardiac computed tomography have substantially expanded the diagnostic capabilities of non-invasive cardiovascular imaging [1,2]. Among these innovations, the development of photon-counting detector CT (PCCT) represents a major technological leap, offering enhanced spatial resolution, improved contrast-to-noise ratio, and intrinsic spectral imaging capabilities compared with conventional energy-integrating detector systems [3,4]. These properties enable a more accurate characterization of coronary arteries [5] and even pericardial or myocardial tissue composition [6,7] and may facilitate the detection of subtle structural alterations associated with various cardiac pathologies and risk factors as a new promising tool for cardiovascular risk analysis [8].

Concurrently, the field of radiomics—the high-throughput extraction and analysis of large sets of quantitative imaging features—has emerged as a promising tool for capturing tissue heterogeneity and subvisual patterns that are generally not analyzed by standard clinical use [9]. When applied to cardiac imaging, radiomics provides a pathway toward objective, reproducible, and data-driven assessment of cardiac tissue characteristics, potentially serving as a bridge between imaging phenotypes and underlying pathophysiology [10,11].

The integration of radiomic analysis with PCCT offers a unique opportunity to leverage the rich spatial and spectral information inherent to this novel imaging technology. By quantifying subtle textural and spectral variations within the myocardium, radiomics may enable improved differentiation between normal and diseased tissue, facilitate early detection of myocardial pathology, and contribute to refined risk stratification and personalized patient management [12].

Cigarette smoking and nicotine exposure are established cardiovascular risk factors and contribute to endothelial dysfunction, oxidative stress, inflammation, and adverse myocardial and vascular remodeling, which may also be associated with increased myocardial contractility and elevated myocardial oxygen demand [13]. These alterations contribute to a higher incidence of adverse cardiovascular events, such as myocardial infarction [14]. Nicotine promotes atherogenesis and hypertensive heart disease, primarily resulting from its hypertensive effects. Consequently, chronic nicotine exposure significantly elevates the risk of cardiovascular morbidity and mortality [15].

Therefore, this study aimed to investigate whether myocardial radiomic features derived from cardiac PCCT are associated with self-reported smoking status in patients without coronary calcification or coronary stenosis. We hypothesized that radiomics may capture subtle myocardial texture differences not reflected by conventional septal thickness measurements.

## 2. Materials and Methods

**Study design and patient cohort:** This single-center retrospective study analyzed 726 patients who underwent clinically induced cardiac PCCT between August 2022 and November 2024 at the University Medical Center Mannheim following the current European Society of Cardiology (ESC) guidelines [16]. Out of 726 patients, 111 patients showed an Agatston score of 0 and no evidence of coronary stenosis or non-calcified plaque on CCTA and were therefore eligible for inclusion. Inclusion criteria comprised patients aged ≥18 years undergoing clinically indicated cardiac PCCT with an Agatston score of 0 and absence of coronary stenosis or non-calcified plaque. Exclusion criteria included insufficient image quality, preventing reliable myocardial segmentation and analysis. Image data were evaluated by a board-certified radiologist with over 10 years of experience in cardiothoracic imaging. After further excluding 7 patients because of insufficient image quality, the study population consisted of 104 patients. Cardiovascular risk factors, including arterial hypertension, diabetes mellitus, dyslipidemia, and smoking status, were assessed using a standardized questionnaire before CT acquisition. Positive smoking status was defined as current active smoking or a prior smoking history of minimum 5 pack-years within 10 years preceding the date of examination. The study was conducted following the principles of the Declaration of Helsinki and obtained approval from both the institutional review board and the local ethics committee (ID 2021-659).

**Figure 1:**
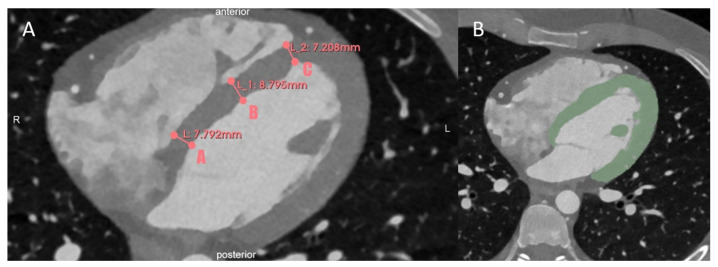
(**A**). Example myocardial septum measurements performed at the apical (A), mid-ventricular (B), and basal (C) septal positions (red lines) (65–70% heart phase). (**B**). Segmentation of myocardial tissue (green area) in a 28-year-old patient.

**CT Imaging Protocol and Analysis:** All patients included received a non-contrast cardiac CT for coronary artery calcification (CAC) evaluation, followed by a contrast-enhanced cardiac CT, both on a first-generation whole-body dual-source PCCT (NAEOTOM Alpha; SiemensHealthcare GmbH, Erlangen, Germany), using a prospective or retrospective ECG-gated scanning mode (depending on heart rate/heart rate variability). Tube voltage was 120 kV, and automatic dose modulation with CARE keV BQ setting of 64. The effective gantry rotation time was 0.25. Patients received up to 15 mg of intravenous metoprolol and up to 0.8 mg sublingual Nitroglycerin as premedication for reducing heart rate and granting vasodilatation. Non-contrast-enhanced cardiac CT (axial reconstruction consisting of 3 mm slice thickness, 3 mm increment, and a Qr36 kernel) was performed for evaluation of CAC using a dedicated software (Syngovia VC10A, Siemens Healthcare GmbH, Erlangen, Germany). Contrast-enhanced CT (axial reconstructions using 0.6 mm slice thickness, 0.4 mm increment and a Bv40 kernel) was performed using 70–80 mL of iodine contrast (Imeron 350, Bracco Imaging Deutschland GmbH, Konstanz, Germany) followed by a 30–40 mL saline chaser (NaCl 0.9%) with a weight-based flow rate of 4.5–6 mL/s through the antecubital venous access. CCTA acquisition was triggered using bolus tracking in the ascending aorta with a threshold of 140 HU at 90 kV. Image data was anonymized, exported and converted to Neuroimaging Informatics Technology Initiative (NIFTI) file format for use with a dedicated segmentation tool (3D Slicer, Version 5.2.2, The Slicer Community, Boston, MA, USA). Myocardial tissue was manually segmented and subsequently reviewed by a board-certified radiologist with 12 years of clinical experience in cardiovascular imaging and 8 years of segmentation expertise. The endocardial and epicardial borders were manually delineated on axial CCTA images, carefully excluding the ventricular blood pool and pericardial fat. Papillary muscles were included in the myocardial mask. Myocardial septal thickness was determined by averaging three measurements in the anterior, mid and posterior parts of the septum on the level of the mitral valve in the 65–70% heart phase. An example segmentation is illustrated in Figure 1.

**Figure 2:**
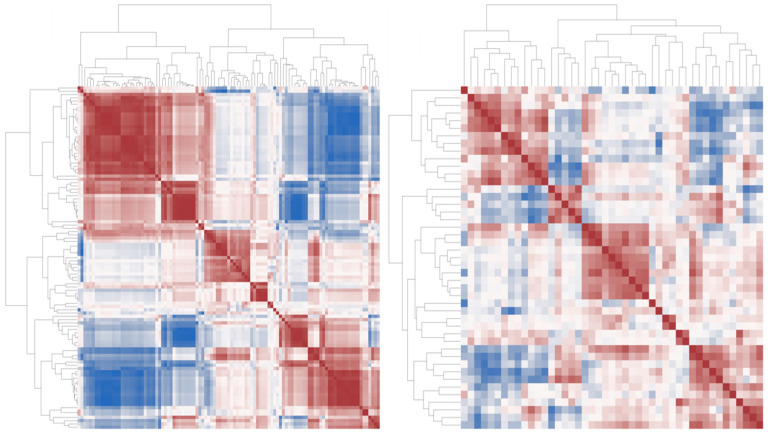
Heatmap of extracted features before (**left**) and after (**right**) reduction with |r| > 0.9 correlation filter. Red color indicates a stronger cluster, while blue indicates a less clustered area. The clear separation of clusters indicates a successful reduction in multicollinearity.

**Radiomics Feature Extraction, Preprocessing, and Statistical Analysis:** Radiomic feature extraction was performed using PyRadiomics version 3.1.0 in Python. Only original image features were extracted, including first-order statistics, shape features, and higher-order texture features derived from GLCM, GLRLM, GLSZM, GLDM, and NGTDM matrices. Feature extraction settings followed the Image Biomarker Standardization Initiative [17] recommendations where applicable. Given the high dimensionality and potential multicollinearity of radiomics features, dimensionality reduction was performed to prevent overfitting. An exploratory correlation analysis using Spearman’s rank correlation was conducted, and a strict threshold of ρ > 0.9 was applied to identify highly correlated feature pairs. Redundant features were systematically removed from the dataset. Correlation matrices before and after feature selection were visualized as heatmaps to assess the effectiveness of redundancy reduction and the independence of remaining features (see Figure 2).

Based on the 45 selected PCCT radiomics features, three machine learning algorithms were trained to predict nicotine status. Due to class imbalance within the dataset (*n* = 104; 67 non-smokers vs. 37 smokers), balanced class weighting (class_weight = balanced) was applied in linear models to reduce bias toward the majority class.

Several machine learning algorithms were trained and evaluated, including logistic regression with L2 regularization, random forest classifiers, and gradient boosting methods. Model performance was evaluated using stratified five-fold cross-validation. To avoid information leakage, feature scaling and correlation-based feature reduction were performed within the training folds of each cross-validation split. The primary evaluation metric was the area under the receiver operating characteristic curve (ROC-AUC). Additional metrics included precision–recall AUC (PR-AUC), balanced accuracy, F1-score, precision, and recall to provide a comprehensive assessment, particularly in the presence of class imbalance. To ensure model interpretability, feature importance was analyzed using model-specific metrics. In particular, logistic regression coefficients were examined to quantify the direction and magnitude of the most influential predictors on classification outcomes.

**Figure 3:**
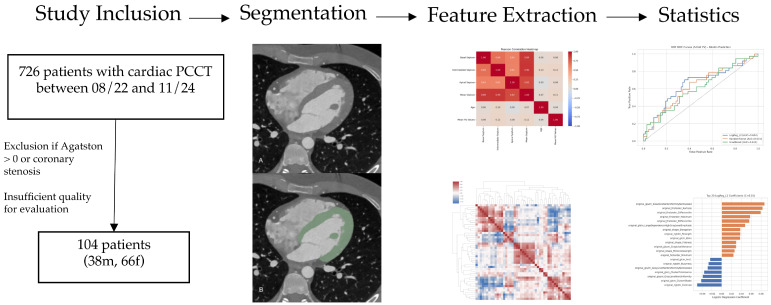
Schematic workflow illustrating study inclusion, myocardial segmentation, radiomics feature extraction, and subsequent statistical analysis. The figure is intended for workflow demonstration purposes only.

For the initial characterization of the study population, descriptive statistics were applied. Continuous variables are reported as mean ± standard deviation (SD), while categorical variables are presented as absolute and relative frequencies. Univariate group comparisons with respect to the primary outcome (nicotine status) were conducted using Welch’s *t*-test or the non-parametric Mann–Whitney U test, depending on data distribution and variance homogeneity. Effect sizes (Cohen’s d and Cliff’s delta) were calculated to quantify the magnitude of observed differences. *p*-values for group comparisons were calculated using either Fisher’s exact test or the Chi-square test, as appropriate. A two-sided *p*-value < 0.05 was considered statistically significant. An overview of the workflow can be seen in Figure 3.

**Statistical Software:** All data processing, statistical analyses, and implementation of machine learning methods were performed using Python (version 3.11). Data preprocessing and management were conducted with pandas (version 2.0.3), while numerical computations were carried out using NumPy (version 1.24.3). Machine learning models were developed, optimized, and evaluated using scikit-learn (version 1.3.0). Visualization of results, including heatmaps and receiver operating characteristic (ROC) curves, was performed using seaborn (version 0.12.2) and matplotlib (version 3.7.1).

## 3. Results

**Patient collective:** A total of 104 patients were enrolled in this study, including 38 men and 66 women, with a median age of 54 years, ranging from 18 to 79 years of age. All patients had no coronary stenosis with a calculated Agatston Score of 0 and a lack of non-calcified plaques in CCTA. 87% of the CT scans were performed in prospective high-pitch or adaptive sequence mode, while about only 13% were acquired retrospectively. A total of 37 patients reported smoking with an average of 16.4 pack-years (16 male, 21 female; median age: 53 years), whereas 67 patients reported non-smoking (22 male, 45 female; median age: 54 years). Available clinical data regarding risk factors are shown in Table 1. Both patient cohorts showed similar distribution of cardiovascular risk factors with no statistically significant difference (*p* > 0.05).

**Figure 4:**
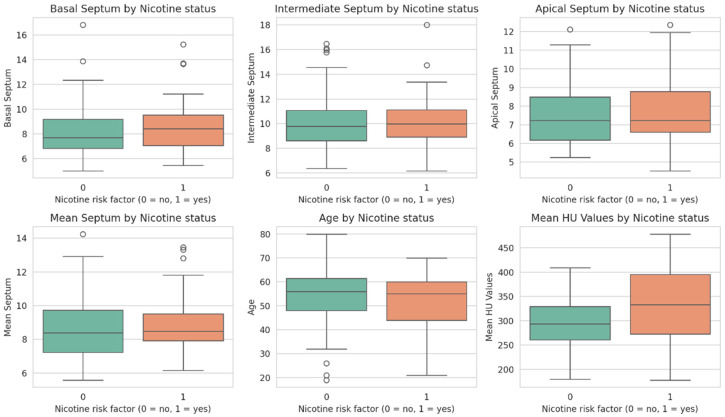
Septum thickness at three different measurement points and age in relation to nicotine status. No significant correlation could be found. Circles represent outliers.

**Univariate analysis of myocardial thickness:** For the univariate analysis, smokers did not exhibit significantly different interventricular septal thickness compared with non-smokers in the basal (8.66 mm vs. 8.30 mm, *p* = 0.307), mid-ventricular (10.27 mm vs. 10.17 mm, *p* = 0.537), or apical segments (7.77 mm vs. 7.46 mm, *p* = 0.592). The individually calculated mean septal thickness was likewise not significantly different between groups (8.90 mm vs. 8.65 mm, *p* = 0.476). As shown in Figure 4, the distributions of septal thickness measurements across all regions display overlapping interquartile ranges and similar medians for smokers and non-smokers.

No significant correlations were found between septal thickness and nicotine status using either point-biserial or Spearman’s rank correlation analyses (mean septal thickness: r = 0.068, *p* = 0.494). In contrast, mean myocardial attenuation values were significantly higher in smokers than in non-smokers (336 HU vs. 298.09 HU; r = 0.275, *p* = 0.005). Age did not differ significantly between groups and was not significantly associated with nicotine status (r = −0.143, *p* = 0.147; see Figure 4).

**Feature Selection, Model Development and Performance Evaluation:** After correlation-based feature reduction, 45 independent radiomics features remained from the initial set of 105 extracted parameters. In direct model comparison, logistic regression with L2 regularization (Log-Reg_L2) demonstrated the highest overall predictive performance. Using out-of-fold predictions as the primary performance metric, this model achieved an area under the receiver operating characteristic curve (ROC-AUC) of 0.658 (95% confidence interval: 0.541–0.769).

**Figure 5:**
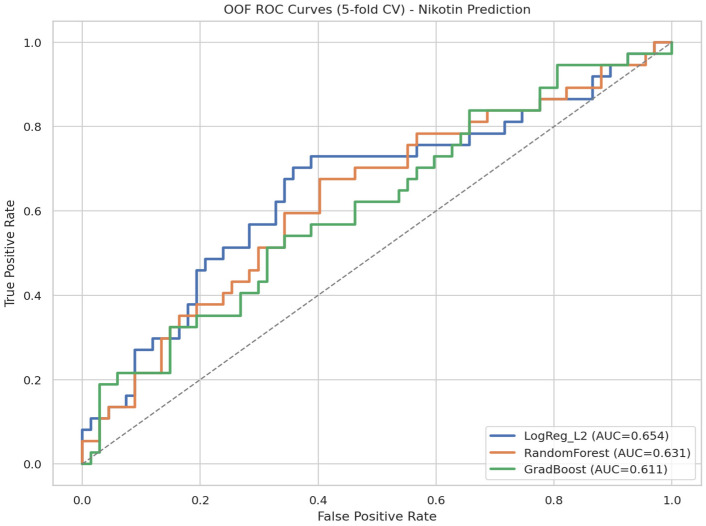
Out-of-Fold (OOF) ROC Curves (with 5-fold cross-valdiation) of three statistical models showing the overall best performance of the Logistic Regression model. The dashed line represents chance-level performance.

Tree-based algorithms showed lower discriminative performance in comparison. The random forest classifier achieved an AUC of 0.630 (95% confidence interval: 0.514–0.741), while the gradient boosting model reached an AUC of 0.609 (95% confidence interval: 0.497–0.719; see Figure 5).

**Figure 6:**
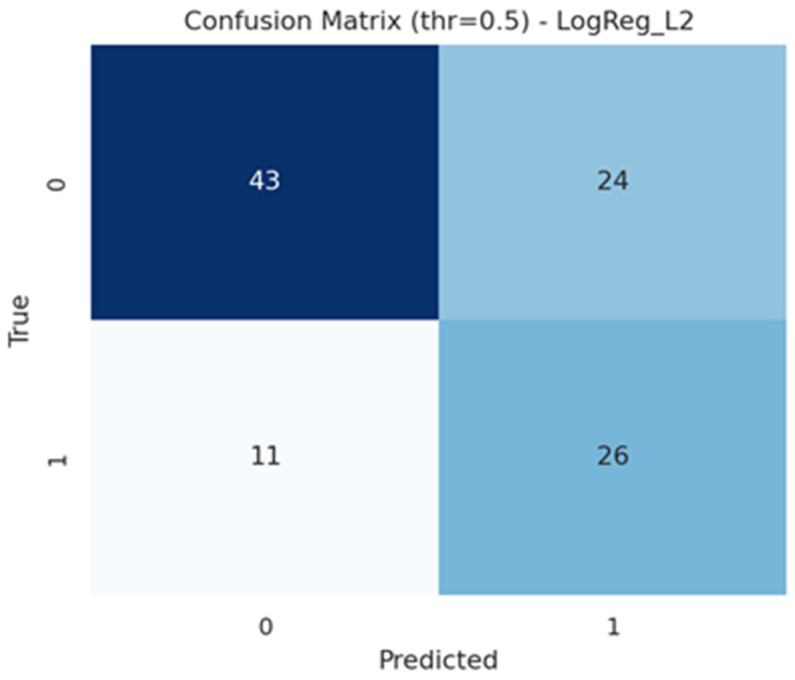
Confusion matrix of Log-Reg_L2 Model. The model correctly classified 43 non-smokers and 26 smokers, with 24 false-positive and 11 false-negative classifications.

When applying a predefined decision threshold, logistic regression demonstrated a balanced trade-off between sensitivity and specificity. Sensitivity reached 70.3% at a specificity of 64.2%, corresponding to the correct identification of 26 smokers (true positives) and 43 non-smokers (true negatives) (see Figure 6). This was reflected in the highest balanced classification accuracy (balanced accuracy = 0.672) and the highest F1-score (0.598) among the evaluated models (see Table 2). 

**Figure 7:**
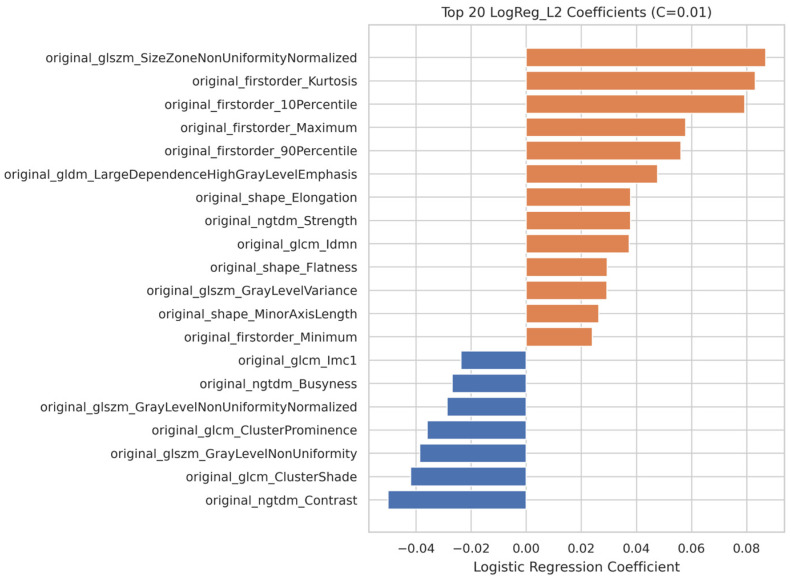
Top 20 features of the Logistic Regression Model with positive (orange color) and negative (blue color) coefficients for nicotine abuse.

In Figure 7, the top 20 features of the L2-regularized logistic regression model can be seen, highlighting the most influential radiomics features for nicotine status prediction. Positive coefficients, such as original_glszm_SizeZoneNonUniformityNormalized—which reflects the variability of homogeneous gray-level zone sizes, with higher values indicating greater non-uniformity of zone size distribution—and original_firstorder_Kurtosis (shape parameter), a first-order feature describing the peakedness of the gray-level intensity distribution, are associated with an increased likelihood of being classified as a smoker, whereas negative coefficients, including original_ngtdm_Contrast (heterogenous voxel gray level) and original_glcm_ClusterShade (reflects local gray-level differences between neighboring voxels and may represent spatial intensity heterogeneity), contribute to the classification of non-smokers. Overall, the magnitude of the coefficients indicates that no single feature dominates the model, but rather that prediction is driven by a combination of multiple texture and shape characteristics.

## 4. Discussion

This study explored whether smoking status is associated with myocardial radiomic texture patterns in patients without coronary calcification or coronary stenosis. While no significant differences in septal thickness were observed between smokers and non-smokers, PCCT-based radiomics enabled moderate discrimination of smoking status, suggesting the presence of subtle myocardial texture patterns associated with smoking exposure, supporting the hypothesis that smoking induces subtle tissue alterations not captured by conventional imaging metrics [18].

Radiomics enables the quantification of spatial tissue heterogeneity beyond simple attenuation values and overcomes limitations of conventional imaging approaches [19,20,21]. In this study, both first-order and higher-order texture features contributed to the discrimination of nicotine status, with machine learning achieving moderate predictive performance (AUC = 0.66). Logistic regression with L2 regularization provided the most robust and interpretable model, consistent with prior radiomics studies emphasizing the value of regularized linear models in high-dimensional datasets [22,23].

From a pathophysiological perspective, these findings align with known nicotine-induced mechanisms, including oxidative stress, endothelial dysfunction, matrix remodeling, and early interstitial fibrosis [24,25,26]. Radiomics analysis appears capable of capturing these subvisual remodeling processes by detecting subtle changes in voxel-level intensity distribution and spatial relationships [27]. Although no histopathological, biomarker-based, or cardiac MRI reference standard was available in the present study, prior radiomics investigations, particularly large CMR-based studies from the UK Biobank, suggest that heterogeneous myocardial texture patterns may represent indirect imaging correlates of early interstitial remodeling and fibrosis. CT-based studies have further demonstrated that myocardial fibrosis and interstitial expansion can be quantified using advanced imaging approaches, supporting the biological plausibility of the observed radiomic alterations [28].

The use of PCCT represents a key technological advancement, enabling improved spatial resolution and reduced noise, thereby enhancing the detection of fine textural patterns [29]. Recent studies demonstrate that PCCT improves the stability and discriminatory power of higher-order radiomics features compared with conventional CT systems [30]. However, radiomic signatures remain modality-specific and may not be directly transferable across imaging platforms [21].

In comparison with prior studies, MRI-based radiomics investigations demonstrate consistent findings, with similar predictive performance and superiority over conventional imaging parameters. Cetin et al. used a large cohort (n = 5065 patients) from the UK Biobank and demonstrated that quantitative texture and shape features provide complementary information beyond conventional volumetric measures such as ventricular size and function. In their analysis, 684 radiomics features were extracted from CMR images and evaluated using machine learning, with L1-regularized logistic regression showing the best performance. Radiomics signatures outperformed conventional imaging metrics in discriminating cardiovascular risk factors (e.g., diabetes AUC 0.80 vs. 0.70), indicating improved sensitivity to subtle tissue alterations. Overall, their findings show that radiomic signatures can discriminate cardiovascular risk factors with meaningful accuracy, suggesting sensitivity to early myocardial remodeling [18,31]. Additionally, emerging CT-based radiomics studies have shown strong diagnostic and prognostic performance in cardiovascular disease, further supporting the clinical relevance of this approach [32,33]. Ayx et al. investigated whether radiomic features derived from PCCT can detect myocardial alterations associated with coronary artery calcification, an established independent risk factor for adverse cardiac events. In this retrospective single-center study of 30 patients, left ventricular myocardium was segmented and analyzed using PyRadiomics, with feature selection performed via random forest. Patients were stratified by Agatston score (0 vs. ≥100), and a subset of four higher-order radiomics features was identified that reliably discriminated between groups, with additional validation showing a severity-dependent trend across intermediate calcium scores. Their findings suggest that increasing coronary calcification is associated with measurable changes in myocardial texture and may serve as a non-invasive biomarker reflecting the burden of coronary artery calcification [34]. While cardiac MRI remains the reference standard for tissue characterization, CT-based radiomics—especially with PCCT—emerges as a promising complementary modality, enabling simultaneous coronary and myocardial assessment without additional imaging [35,36,37].

Despite these encouraging results, several limitations must be acknowledged. First, smoking status was self-reported and only limited information was available on pack-years, smoking duration, former smoking status, passive smoke exposure, or the use of nicotine replacement therapy, which may have led to misclassification and residual confounding. Second, although dimensionality reduction and cross-validation techniques were applied, the relatively small cohort size compared with the number of retained radiomics features may still predispose the models to residual overfitting and reduced stability [22]. Consequently, the modest discriminatory performance observed (AUC = 0.66) should be interpreted cautiously, and larger multicenter studies with external validation are required to confirm the robustness and generalizability of the identified radiomic signatures. Third, coronary artery disease exclusion was based on non-invasive CCTA rather than invasive coronary angiography, which remains the reference standard, although CCTA provides excellent diagnostic accuracy and high negative predictive value for excluding obstructive coronary artery disease [38].

Furthermore, radiomics features are known to be sensitive to variations in image acquisition and reconstruction protocols, which may affect their robustness and reproducibility across different settings. In addition, no histopathological, cardiac magnetic resonance, or functional reference standard was available to confirm whether the observed texture differences truly reflect underlying tissue characteristics such as fibrosis, inflammation, or edema. Finally, the retrospective cross-sectional design precludes causal inference and limits conclusions to associations rather than cause–effect relationships.

## 5. Conclusions

In this exploratory single-center study, PCCT-based myocardial radiomics showed moderate ability to discriminate patients according to smoking status, whereas conventional septal thickness measurements did not differ between groups. These findings suggest that radiomic texture features may capture subtle imaging patterns associated with smoking exposure. Larger prospective studies with quantitative smoking exposure assessment, standardized radiomics workflows, multimodal tissue correlation, and external validation are warranted. Further technical refinement and integration with clinical and imaging biomarkers may improve the robustness and clinical applicability of cardiac radiomics for early cardiovascular risk stratification.

## Figures and Tables

**Table 1:** Comparison of different cardiovascular risk factors (diabetes, dyslipidemia, and hypertension) for patients with and without nicotine abuse.

Risk Factor	Nicotine + (n = 37)	Nicotine − (n = 67)	*p*-Value
Diabetes	5 (14%)	4 (6%)	0.21
Dyslipidemia	9 (24%)	18 (27%)	0.75
Hypertension	18 (49%)	30 (45%)	0.7

**Table 2:** Statistical performances with Area under the curve (AUC), Accuracy values and F1-Scores of three machine learning models.

Model	ROC-AUC	Accuracy	F1-Score
Logistic Regression	0.658 (0.541–0.769)	0.672	0.598
Random Forest	0.630 (0.514–0.741)	0.582	0.451
Gradient Boosting	0.609 (0.497–0.719)	0.541	0.382

## Data Availability

The data presented in this study are available upon request from the corresponding author but are not publicly available due to privacy policy restrictions.

## Abbreviations

The following abbreviations are used in this manuscript:

ACS	Acute coronary syndrome
AUC-ROC	Area under the ROC curve
Bv40	Body vascular reconstruction kernel
CAC	Coronary artery calcification
CAD-RADS	Coronary artery disease reporting and data system
CCTA	Coronary computed tomography angiography
CNR	Contrast-to-noise ratio
CT	Computed tomography
CTA	Computed tomography angiography
CVD	Cardiovascular disease
ECG	Electrocardiogram
EID	Energy-integrating detector
ESC	European Society of Cardiology
GB	Gradient Boosting
HU	Hounsfield Units
LoG	Laplacian of Gaussian
Log Reg	Logistic Regression
MRI	Magnetic resonance imaging
NPV	Negative predictive values
PCCT	Photon-counting computed tomography
PPV	Positive predictive values
RF	Random Forest
ROI	Region of interest
ROC	Receiver operating characteristic curve
SD	Standard deviation
Qr36	Photon-counting body vascular reconstruction kernel
